# Mechanisms of Exogenous L-Lysine in Influencing the Quality of Low-Sodium Marinated Braised Beef

**DOI:** 10.3390/foods14132302

**Published:** 2025-06-28

**Authors:** Chongxian Zheng, Pengsen Wang, Mingming Huang, Tong Jiang, Jianying Zhao, Yanwei Mao, Huixin Zuo

**Affiliations:** 1 College of Food Science and Engineering, Shandong Agricultural University, Tai’an 271018, China; 17661291657@163.com (C.Z.); sdauwps@163.com (P.W.); hades3709@126.com (M.H.); 13064653588@163.com (T.J.); maoyanwei@sdau.edu.cn (Y.M.); 2 College of Tea and Food Technology, Jiangsu Vocational College of Agriculture and Forestry, Jurong 212400, China; callfull@163.com

**Keywords:** salt reduction, meat quality, beef processing, amino acid, product storage

## Abstract

During the processing of marinated braised beef, excessive sodium intake is likely to occur, which can lead to various health issues. Exogenous L-lysine (L-Lys), as an essential amino acid for the human body, has the capability to enhance the quality of low-sodium meat products. This study aimed to investigate the effects of exogenous L-Lys on the quality of low-sodium plain boiled beef and marinated braised beef, as well as its underlying mechanisms of action. Among them, the substitution rate of KCl was 60%. This study was conducted with three batches of experiments, each batch serving as an independent parallel. For low-sodium plain boiled beef, the optimal addition level of L-Lys was screened out through the research on the effects on meat quality indicators, water distribution, microstructure, and sensory evaluation. For the quality of low-sodium plain boiled beef, in terms of microstructure, the addition of L-Lys reduced muscle fiber breakage and voids, thereby improving its microstructural characteristics. Combined with quantitative descriptive analysis (QDA), the optimal level of additional L-Lys was subsequently determined to be 0.6%. It was further processed into marinated braised beef in soy sauce, and a comparative analysis was conducted with low-sodium marinated braised beef in soy sauce without L-Lys addition for shear force, meat color, thiobarbituric acid reactive substances (TBARS), and total viable count (TVC) during the storage periods of 0, 3, 6, 9, and 12 d. The results show that the redness (a*) value significantly increased within 0–12 d (*p* < 0.05), leading to a more stable meat color. Moreover, the addition of L-Lys significantly reduced the shear force and thiobarbituric acid reactive species (TBARS) values in the marinated braised beef (*p* < 0.05), thereby optimizing the tenderness of the marinated braised beef and inhibiting lipid oxidation. Although the total viable count (TVC) of the L-Lys group was higher than that of conventional low-sodium marinated braised beef in soy sauce from 9 to 12 d, both groups of products had undergone spoilage by day 12; therefore, the addition of L-Lys had no effect on the shelf life of the products. Comprehensive analysis suggested that the addition of exogenous L-Lys could optimize beef quality by enhancing hydration, improving muscle structural properties, and exerting antioxidant synergistic effects.

## 1. Introduction

Plain boiled beef, as a high-protein and low-fat meat product, is favored by consumers, according to Bhat et al. [1]. However, during the processing of plain boiled beef, issues such as meat texture hardening, juice loss, and flavor deterioration often occur, especially under low-sodium conditions. The development of low-sodium foods represents a crucial strategy to address health problems caused by modern high-sodium diets, such as hypertension and cardiovascular diseases. Nevertheless, the reduction in sodium salts typically leads to the deterioration of water-holding capacity (WHC), texture, and flavor in meat products, as described by Inguglia et al. [2].

The reduction in sodium in traditional high-salt processing methods could lead to decreased protein solubility, increased moisture loss, disruption of myofibrillar structure, and dull coloration of the product, as reported by Zhang et al. [3]. These issues severely restrict the quality and market competitiveness of the products. Currently, the primary sodium chloride substitution technologies involve utilizing other metal salts with properties similar to those of NaCl to replace sodium chloride, thereby reducing sodium intake. Among them, the main substitutes for NaCl include KCl, MgCl_2_, CaCl_2_, phosphates, etc. [4,5]. However, the substitution of NaCl with other metal salts is not without drawbacks. When the replacement ratio of NaCl reaches a certain level, the products tend to develop metallic and bitter flavors, while the intensity of saltiness diminishes. These changes could severely compromise the flavor profile of meat products [6]. In the low-sodium substitution of meat products, phosphates are the most widely applied by Thangavelu et al. [7], in the marination solution for chicken breast meat, the addition of sodium tripolyphosphate significantly reduced the usage of sodium NaCl. However, excessive intake of phosphorus also poses safety risks to human health, disrupting the elemental balance within the body, particularly imposing a significant burden on kidney function [8,9]. Nowadays, the application of ultra-high pressure (UHP) and ultrasonic technologies in the food sector has become increasingly mature. However, advanced techniques, such as UHP treatment or vacuum packaging, entail high costs and pose significant challenges for industrial-scale implementation, as reported by Li et al. [10].

In recent years, the application of exogenous L-lysine (L-Lys) in food processing garnered increasing attention [11]. Numerous scholars successively suggested that basic amino acids could significantly increase the solubility of myosin under low ionic strength. Specifically, the solubility of myosin in environments with low ionic strength was capable of being enhanced by histidine (His) and L-Lys [12]. Among them, exogenous L-Lys, as an essential amino acid for humans and a multifunctional regulator, was widely recognized as the “first essential amino acid”, as reported by Guo et al. [13]. Owing to its ability to promote protein solubility, enhance hydration, and exhibit antioxidant properties, it emerged as a potential agent for improving the quality of low-sodium meat products [14,15]. Zhang et al. [3] observed that whether used individually or in combination, L-Lys and arginine (Arg) have been shown to enhance the tenderness and WHC of cooked chicken breast meat. Moreover, while L-Lys itself exhibits a certain degree of saltiness, its combination with salt substitutes in a specific proportion could neutralize the bitter and astringent tastes of other metal salts, simultaneously enhancing the saltiness of the mixed salts. Da Silva et al. [16] found that when the substitution ratio of KCl for NaCl reaches 60%, it impairs the emulsion stability, texture structure, and sensory quality of sausage products. Thus, they combined His and Arg, which share similar properties with L-Lys, with the KCl substitution technology. The results indicate that both individual and combined additions of Arg and His effectively mitigated the sensory defects caused by the addition of KCl and enhanced the overall quality of the products [16]. However, current research on L-Lys has not clearly defined its optimal addition level or storage stability, and there is a lack of integrated analysis of the multidimensional effects of L-Lys. It is hypothesized that there exists an appropriate concentration which can enhance the quality, appearance, and microstructure of low-sodium plain boiled beef, and meanwhile moderately improve the edible quality of low-sodium marinated braised beef without affecting the product shelf life. This study aimed to reveal the effects of exogenous L-Lys on the quality indices, distribution of water content, microstructure, and sensory evaluation of plain boiled beef, screen for the optimal addition dosage, further explore the quality and storage stability of marinated braised beef after sauce-braising processing, and integrate the findings from both plain boiled beef and marinated braised beef to provide a theoretical basis and new directions for the development of low-sodium meat products.

## 2. Materials and Methods

### 2.1. Raw Material Preparation

The raw meat used in this experiment was obtained from the beef shank of Simmental × Luxi hybrid cattle provided by a halal meat food company in Heze, Shandong Province. Six cattle with a similar feeding environment and carcass weight of about 350 kg were selected and slaughtered using Islamic slaughtering methods. After slaughter, the carcasses were aged at 4 °C for 24 h, followed by primary segmentation. The segmented meat was transported back to the laboratory at 4 °C for further trimming and then frozen and stored at −20 °C. The pH of the aged beef was measured using a portable pH meter to ensure that the pH values of the selected meat samples ranged between 5.4 and 5.8.

### 2.2. Experimental Design

The beef shanks from the left and right carcasses were trimmed and divided into beef blocks weighing approximately 150 g each, which were then stored at −20 °C. For the experiment, 20 beef blocks were randomly selected and thawed. Then, they were injected with different L-Lys-containing marinades. The injection amount was 20% of the meat weight, and the marinades had a salt concentration of 7.5%, a 60% KCl substitution ratio, 10 h of marination time, and 2 h of cooking time, with the L-Lys addition level serving as the variable. After marination, the beef samples were vacuum-packaged (specifications: 320 mm × 200 mm multilayer heat-shrinkable packaging with high barrier to oxygen (35 cm^3^/m^2^/d at 23 °C) and water vapor (9 gH_2_O/m^2^/d at 38 °C, 90% RH), thickness: 55 μm) and cooked using a water bath method to ensure even heat distribution, then the beef samples were cooled to room temperature and stored overnight at 4 °C. The effects of the different concentrations of L-Lys addition on the quality of low-sodium plain boiled beef were subsequently analyzed.

The remaining beef shanks were divided into beef blocks weighing approximately 150 g each and stored at −20 °C. During the experiment, they were taken out and thawed. The meat pieces obtained from each biceped femoris muscle were randomly grouped. They were, respectively, injected with a brine solution containing 0% (control, C) treatment and a brine solution containing 0.6% (L-Lys, L) treatment of L-Lys for marinating. After the marinating was completed, the marinated samples were braised in a double-jacketed pot for 2 h. The ratio of the braising liquid components was water accounting for 300% of the meat mass, including NaCl 1.2%, KCl 1.8%, broad bean paste 0.7%, monosodium glutamate 0.3%, cooking wine 1%, sucrose 1%, soy sauce 1.7%, bay leaves 0.1%, Angelica dahurica 0.1%, tsaoko fruit 0.1%, fennel 0.1%, cassia bark 0.2%, star anise 0.2%, clove 0.05%, scallions 1.4%, and ginger 1.4%. Then, it was cooled to room temperature. Ten groups were selected and exposed to air for contamination. After that, they were packaged in trays and stored at 4 °C. The storage indexes were determined on day 0, 3, 6, 9, and 12, and the meat color and tenderness were measured. Each piece of meat was measured three times.

### 2.3. Determination of Quality Indicators of Low-Sodium Plain Boiled Beef

#### 2.3.1. pH Value

The pH measurement method was adapted from that reported by Zuo et al. [17]. The pH value was determined using a portable solid-state pH meter (SenVengo, Mettler Toledo, Zurich, Switzerland). The pH meter was calibrated using a two-point method with pH buffers of 4.00 and 7.00, ensuring a slope correction of over 95%. After the low-sodium plain boiled beef was prepared, it was cut in half against the fiber direction using a dissection knife. Six points were randomly and evenly selected from the center of the beef. A small hole was punctured at each selected point using the dissection knife, and the probe was inserted into the hole for measurement. The mean value of the 6 measurements was taken as the final pH value.

#### 2.3.2. Cooking Loss

The method for determining the cooking loss was adapted from that described by Liu et al. [18], with minor modifications. Before cooking, beef samples were dried by blotting surface juices with absorbent paper and weighed as W_1_. The experimental samples were then treated with different marinades, each injected at 20% of the meat weight. After the low-sodium plain boiled beef was prepared, the juices from the cooking bags were drained and the samples were left to cool at room temperature. Once the samples cooled to room temperature, they were further chilled at 4 °C for 12 h. After chilling, the surface juices and any residual blood were blotted dry with filter paper, and the samples were weighed again, with the final weight recorded as W_2_.

Equation (1) is as follows:

(1) $$Cooking\;loss\left(\%\right)=\frac{W_{1}-W_{2}}{W_{1}}\;\times \;100\%.$$

#### 2.3.3. Shear Force

The method for determining shear force was adapted from that reported by Hou et al. [19], with minor modifications. After cooking, low-sodium plain boiled beef samples were cooled to room temperature and stored at 4 °C for at least 12 h. Using a 1.27 cm-diameter hollow corer, cylindrical meat cores longer than 3 cm were cored along the muscle fiber direction, with at least 6 cores collected (at least 2 cores per meat piece), avoiding tendons and fat. Shear force measurements were performed on these cores using a TA-XT2i texture analyzer (TA-XT 2i Stable Micro Systems, London, UK). The average shear force value of the 6 cores was taken as the shear force for that group. For the texture analyzer, specific operation parameters were established. The speed before the test started was 2.0 mm/s. During the testing process, the speed was 1.0 mm/s, and after the test, the speed reached 5.0 mm/s. The force for detection was maintained at less than 1 N. A trigger force of 0.098 N was set to initiate the measurement. The probe was set to penetrate the sample to a distance of 23.0 mm. Additionally, the HDP/BSW type of probe was chosen for conducting the texture analysis.

#### 2.3.4. Meat Color

The method for color measurement was adapted from that described by Zuo et al. [17], with minor modifications. The low-sodium plain boiled beef was cut in half against the fiber direction. The spectrophotometer was calibrated with a white ceramic plate adjusted to Y-93.7, x = 0.3160, and y = 0.3323. A colorimeter (SP62, X-rite, Co., Ltd., Grand Rapids, MI, USA) with a measurement aperture of 8 mm, light source A, and a standard viewing angle of 10° was used to measure the lightness (L*), redness (a*), yellowness (b*), chroma (C*), and hue (H*) values of the beef. The calculation formulas are as follows: C* = (a^*2^ + b^*2^)^1/2^, H* = [arctangent (b*/a*)]. Six random and evenly distributed points were selected on each sample for measurement. The mean value of these 6 points was taken as the final value for each color parameter.

#### 2.3.5. Appearance

After the low-sodium plain boiled beef was prepared, it was cooled to room temperature and then stored at 4 °C for more than 12 h. Suitable samples were selected, and slices approximately 3 mm thick were cut from the middle section of the samples. These slices were placed on a white porcelain plate and illuminated with a white light source at an appropriate brightness level for photography.

#### 2.3.6. Low-Field Nuclear Magnetic Resonance (LF-NMR) and Magnetic Resonance Imaging (MRI)

From the central portion of low-sodium plain boiled beef, 1 cm-thick meat blocks were cut, avoiding tendons and adipose tissues, and further trimmed into 1 cm × 1 cm × 2 cm cylindrical cores along the muscle fiber direction, with at least 3 cores prepared per group. The meat cylinders were gently placed into NMR tubes without compression and sealed with parafilm to prevent moisture evaporation. The LF-NMR spectrometer (NMRI20-015V-I, Niumag Co., Ltd., Shanghai, China) was preheated for 30 min at a test temperature of 32 °C. Prior to testing, the instrument was calibrated using a standard oil sample with a free induction decay (FID) sequence. The samples were measured using the Carr–Purcell–Meiboom–Gill (CPMG) sequence. For MRI testing at 32 °C, the instrument was calibrated with a standard oil sample after preheating, and appropriate images were selected and saved following a pre-imaging process.

#### 2.3.7. Microstructure

The method for determining the microstructure was adapted from that described by Zhang et al. [20], with minor modifications. The microstructure was observed using the scanning electron microscope (SEM). After cooking, low-sodium plain boiled beef samples were cooled to room temperature and stored at 4 °C for over 12 h. From the central portion of each group of low-sodium plain boiled beef, 1 cm × 1 cm × 1 cm meat cubes were excised using a dissecting knife. The samples were subjected to critical point drying followed by gold sputtering. Scanning was performed from 2 orientations, with 4 scans conducted for each orientation. After scanning, appropriate magnifications were selected for analysis. Electron microscopy parameters: accelerating voltage of 3 kV or 5 kV, resolution of 50 nm.

#### 2.3.8. Quantitative Descriptive Analysis (QDA) Evaluation

This study recruited 15 beef quality researchers aged 20–30 years with experience in sensory evaluation. The QDA method was employed to assess the effects of different L-Lys addition levels on the eating quality of plain boiled beef. Samples were thinly sliced into 2–3 mm thickness, randomly coded with 3 slices per person per group, and presented to evaluators under blind testing conditions. Evaluators scored 6 attributes, including color, texture, and tenderness, on a 10-point scale. The entire testing process was conducted in a standardized environment, with taste interference controlled by soda water and soda biscuits. Samples from each treatment group were presented 3 times in a cross-randomized manner to ensure data reliability.

### 2.4. Determination of Storage Indices for Low-Sodium Marinated Braised Beef

#### 2.4.1. Shear Force During Storage

After cooking the 2 groups of low-sodium marinated braised beef, each piece was measured at least 3 times on day 0, 3, 6, 9, and 12 with the specific methods described in Section 2.3.3.

#### 2.4.2. Meat Color During Storage

After cooking the 2 groups of low-sodium marinated braised beef, each piece was measured at least 3 times on day 0, 3, 6, 9, and 12 with the specific methods described in Section 2.3.4.

#### 2.4.3. Thiobarbituric Acid Reactive Substances (TBARS)

A 0.06 mol/L 2-thiobarbituric acid (TBA) solution and a 10% trichloroacetic acid (TCA) solution were prepared in advance, and a standard curve was constructed. The 2 groups of low-sodium marinated braised beef were sampled on day 0, 3, 6, 9, and 12 with 5 replicates per group, respectively. For each replicate, 1 g of sample was collected. Samples were placed into 5 mL grinding tubes, followed by the addition of 4 mL of pure water and steel beads. The mixtures were ground using a freeze grinder at 50 Hz for 45 s, with 3 grinding cycles performed to ensure uniform disruption. Following the grinding process, the samples were transferred into centrifuge tubes with a capacity of 10 mL. Subsequently, 4 mL of the TCA solution was added and the mixture was thoroughly mixed by vigorous shaking. After the shaking was completed, the mixture was filtered using a funnel and filter paper. In total, 1 milliliter of the filtrate was then transferred into a 1.5 mL centrifuge tube, and 0.25 mL of the TBA solution was added. The mixture was subjected to a metal bath at 80 °C for 90 min. After the reaction was completed, 250 μL of the resulting solution was transferred into a microplate, and this procedure was repeated 3 times. The absorbance was measured at 532 nm using a microplate reader. For the blank group, the mixture consisted of 0.5 mL of water, 0.5 mL of TCA, and 0.25 mL of TBA. A standard curve was constructed using solutions of 1,1,3,3-tetraethoxypropane (TEP) at concentrations of 0, 0.05, 0.1, 0.15, 0.2, 0.3, 0.4, 0.6, 0.8, and 1 μmol/L, and the content of malondialdehyde (MDA) was calculated based on this standard curve.

#### 2.4.4. Total Viable Count (TVC)

After the 2 groups of low-sodium marinated braised beef were prepared, on days 0, 3, 6, 9, and 12, 10 g of beef samples were aseptically taken from the surface of the marinated braised beef and placed into stomacher bags. Subsequently, 90 mL of physiological saline containing 0.85% NaCl and 0.1% peptone was added to each bag. The bags were then inserted into a stomacher machine and subjected to mechanical stomaching for 2 min. After stomaching, 1 mL of the original bacterial suspension was successively added to test tubes containing 9 mL of sterile saline (0.85% NaCl, 0.1% peptone) for serial dilution, with vigorous mixing after each addition. One milliliter of the appropriately diluted bacterial suspension was then inoculated onto Petri dishes, to which an adequate amount of plate count agar medium was added and spread evenly using a rotating motion. After the medium was evenly distributed, the Petri dishes were closed and incubated. Three replicates were prepared for each dilution. After 48 h of incubation, the TVC was determined by counting the colonies.

### 2.5. Statistical Analysis of Data

In this study, the data were subjected to significance analysis using SAS 9.0 software. In the single-factor experiment of L-Lys addition, only the level of L-Lys addition was varied. Six cattle were used for biological replication as the random factor, and a single-factor significance analysis was conducted. An appropriate level of L-Lys addition was selected based on the results, which was then used as the data group for the subsequent two-factor experiment. In the two-factor experiment, the addition of L-Lys and storage days were treated as fixed factors, while biological replication was used as the random factor. Analysis of variance (ANOVA) for each indicator was conducted using the mixed model in SAS, with a significance level set at (*p* < 0.05). Graphical analysis was then performed using Origin2024 software based on the significance results.

## 3. Results

### 3.1. The Effects of L-Lys on Low-Sodium Plain Boiled Beef

#### 3.1.1. The Effects of Exogenous L-Lys Addition on the Quality of Plain Boiled Beef

As shown in Table 1, significant differences (*p* < 0.05) were observed in the pH value, cooking loss, shear force, and meat color of low-sodium plain boiled beef with different levels of L-Lys addition. Specifically, with the increased exogenous L-Lys addition levels, the pH value of low-sodium plain boiled beef increased significantly (*p* < 0.05). Additionally, an increase in pH led to an increase in protein net charge and enhanced WHC, as reported by Fu et al. [21], thereby reducing the cooking loss of beef (*p* < 0.05). As the exogenous L-Lys addition levels increased gradually, the shear force of low-sodium plain boiled beef significantly decreased (*p* < 0.05).

**Figure 1:**
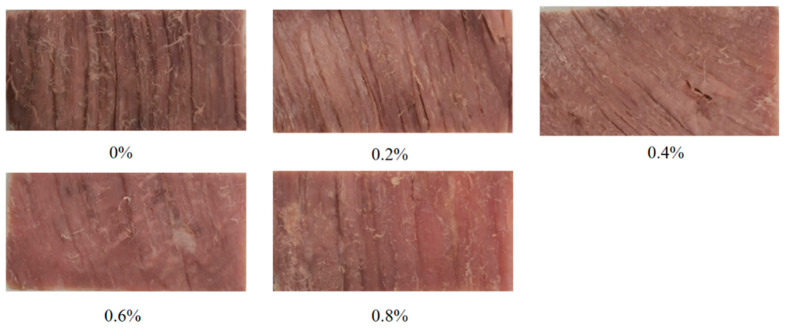
Different L-lysine additions on the appearance of low-sodium plain boiled beef. Note: 0–0.8% of exogenous L-Lys addition was calculated based on meat weight.

As shown in Figure 1, schematic images of the central regions of low-sodium plain boiled beef were presented for exogenous L-Lys addition levels of 0, 0.2, 0.4, 0.6, and 0.8%. The results shown in Table 1 indicate that different L-Lys addition levels had significant effects on the L*, a*, and b* values of low-sodium plain boiled beef (*p* < 0.05). As the L-Lys addition level increased in 0.2% gradients, the a* value significantly increased (*p* < 0.05), while the L* and b* values exhibited a negative correlation with the addition level, with significant overall differences (*p* < 0.05). Moreover, with the addition of exogenous L-Lys, the C* of low-sodium plain boiled beef showed an overall increasing trend, while the H* and W decreased significantly (*p* < 0.05).

#### 3.1.2. The Effects of Exogenous L-Lys Addition on LF-NMR Imaging of Plain Boiled Beef

**Figure 2:**
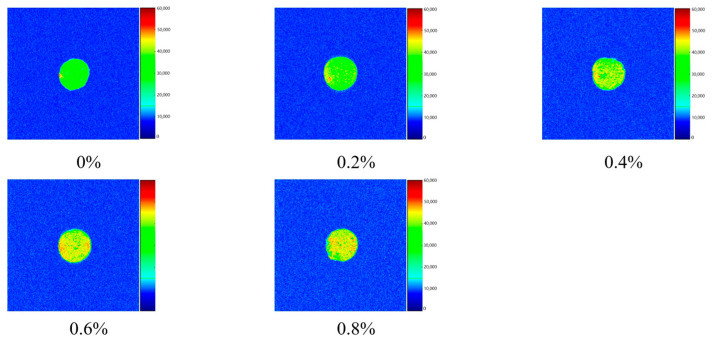
Different L-Lys additions on the magnetic imaging of low-sodium plain boiled beef.

In the results from MRI imaging, a higher number of red dots indicate a higher density of hydrogen (H) protons in the low-sodium plain boiled beef, which corresponds to a higher water content. Conversely, a higher number of green points indicates a lower density of H protons and lower moisture content, with yellow points representing an intermediate state between green and red points. As shown in Table 2 and Figure 2, as the L-Lys addition level increased, the *T*_21_ peak showed an overall downward trend. The *T*_22_ peak significantly increased when the addition level ranged from 0 to 0.8% (*p* < 0.05), but significantly decreased from the 0.2% to 0.8% addition levels (*p* < 0.05). Meanwhile, the increasing number of red points in LF-NMR imaging with higher L-Lys addition levels indicated a transformation of free water into bound and immobilized water, which was consistent with the trend of cooking loss and correlated with L-Lys-promoted actomyosin dissociation and improved WHC.

#### 3.1.3. Exogenous L-Lys Addition on the Microstructure of Plain Boiled Beef

**Figure 3:**
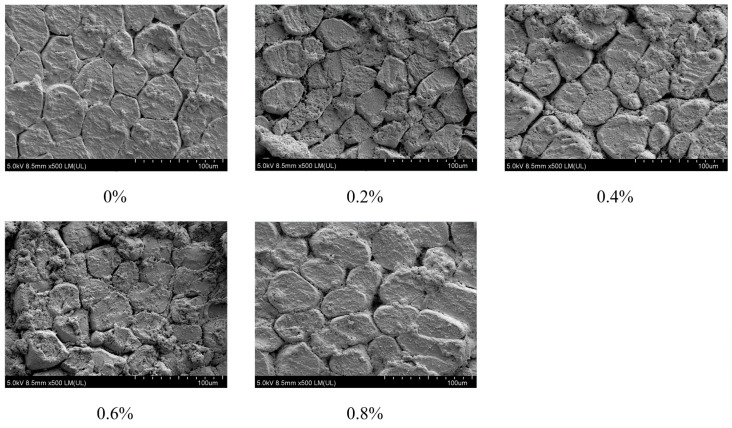
Different L-Lys content on microstructure of low-sodium plain boiled beef.

Figure 3 shows the effect of different addition amounts of exogenous L-Lys on the microstructure of plain boiled beef. As shown in Figure 3, when the addition amount is 0–0.2%, the muscle structure begins to show slight loosening, and the fiber gap slightly increases, but the muscle fibers still maintain a relatively large continuity. When the addition amount reaches 0.6%, the muscle fibers are significantly dissociated, forming a uniformly dispersed reticular structure with a marked increase in the number and size of pores, indicating a well-formed gel structure.

#### 3.1.4. QDA of Low-Sodium Plain Boiled Beef

**Figure 4:**
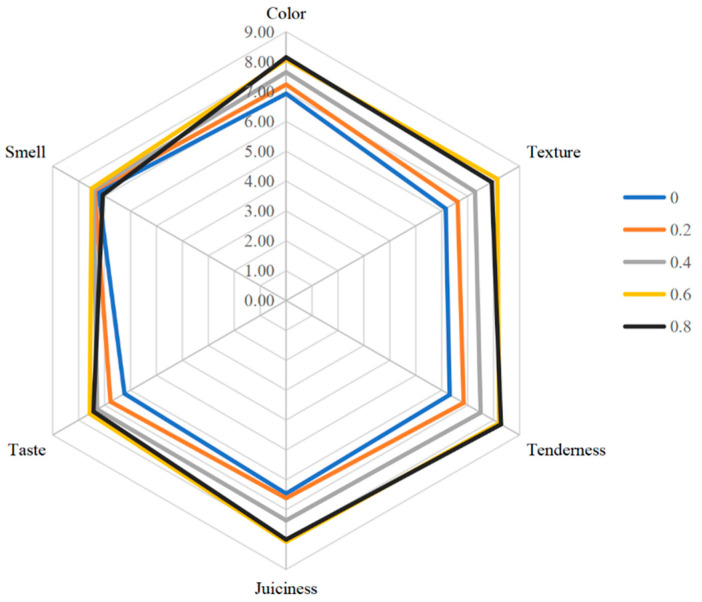
Different L-Lys addition levels on QDA evaluation of low-sodium plain boiled beef.

As shown in Figure 4, with the increase in L-Lys addition, the sensory evaluation of low-sodium plain boiled beef continuously improved. The scores for each index significantly increase within the range of 0–0.6% addition; however, when the addition reaches 0.8%, the low-sodium plain boiled beef begins to exhibit certain off-flavors, leading to a decrease in the score. Additionally, no significant improvement is observed in other indicators, resulting in a reduction in the total score. Therefore, the sensory evaluation score of low-sodium plain boiled beef is highest when the addition of L-Lys is 0.6%.

#### 3.1.5. Exogenous L-Lys Correlation Analysis

**Figure 5:**
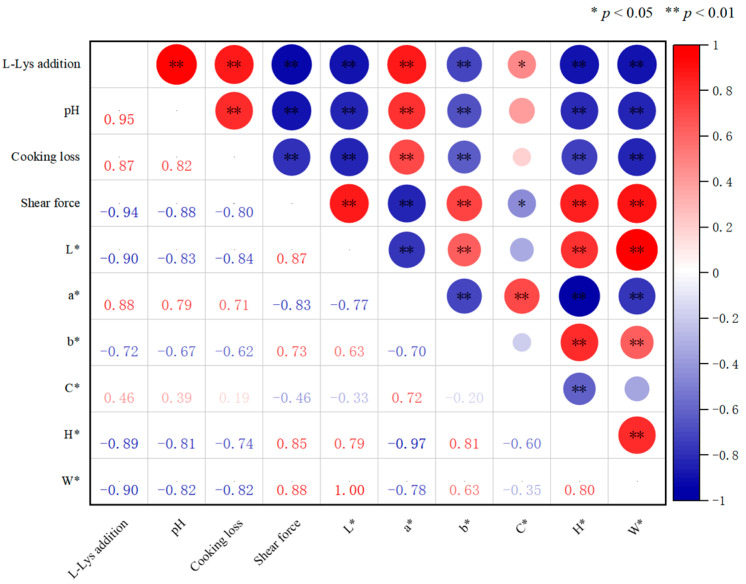
Different L-Lys addition levels on correlation analysis of low-sodium plain boiled beef.

As shown in Figure 5, the addition of exogenous L-Lys was extremely significantly positively correlated with pH (*p* < 0.05), indicating that the increase in the amount of exogenous L-Lys added promoted the rise of pH in low-sodium plain boiled beef, affecting the basic chemical environment of meat products. It had an extremely significant negative correlation of 0.82 with cooking loss (*p* < 0.05). The increase in L-Lys addition effectively reduced the loss of moisture and other substances during cooking, enhancing the water-holding capacity of low-sodium plain boiled beef. Meanwhile, the addition of L-Lys had a strong negative correlation with shear force (*p* < 0.05), suggesting that the addition of exogenous L-Lys could reduce the shear force of low-sodium plain boiled beef and enhance its tenderness. In terms of meat color correlation, the addition of L-Lys had an extremely significant positive correlation of 0.88 with a*, a positive correlation with C*, and negative correlations with L*, b*, H*, and W* (*p* < 0.05), making the color more bright red and natural.

In conclusion, exogenous L-Lys plays a crucial role in enhancing the quality of low-sodium plain boiled beef. The incorporation of L-Lys significantly improved the WHC of low-sodium plain boiled beef, reduced its cooking loss rate, decreased shear force, refined microstructure, and enhanced color stability, appearance quality, and flavor. Combined with the QDA sensory evaluation, when the addition amount was 0.8%, the low-sodium plain boiled beef produced a strong unpleasant odor, which seriously affected the edible quality. Comprehensive analysis showed that when the L-Lys addition amount was 0.6%, the improvement effect on low-sodium plain boiled beef was the best. The group with 0.6% L-Lys addition (L group) was selected as the optimal treatment for further processing into braised beef. This group was compared with low-sodium marinated braised beef without L-Lys addition (C group) to investigate the effects of L-Lys on beef quality during processing and storage.

### 3.2. The Effects of L-Lys on Low-Sodium Marinated Braised Beef

#### 3.2.1. Exogenous L-Lys Addition on Tenderness of Low-Sodium Marinated Braised Beef

**Figure 6:**
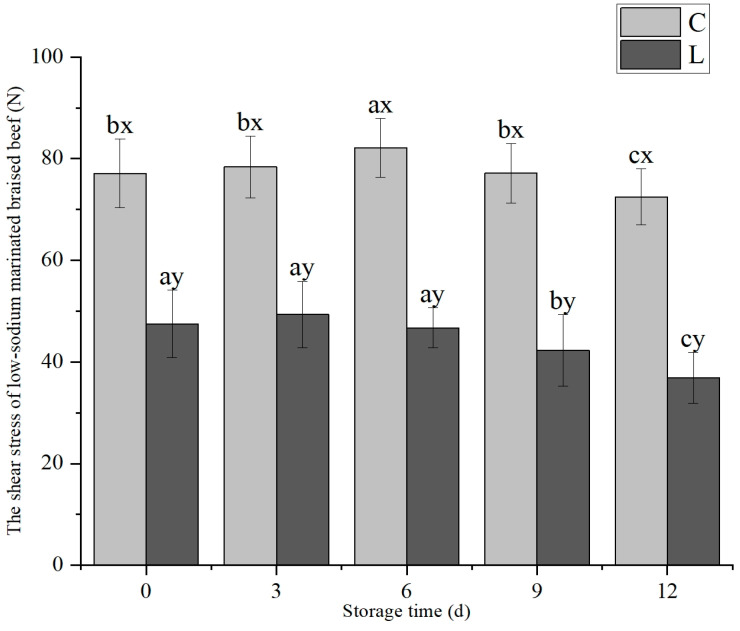
Exogenous L-Lys on shear stress changes of low-sodium marinated braised beef during storage. The letters ^a–c^ indicate significant differences (*p* < 0.05) among different storage times for the same exogenous L-Lys treatment. The letters ^x,y^ denote significant differences (*p* < 0.05) among different exogenous L-Lys treatments at the same storage time.

The addition of lysine and storage time both had significant effects on the shear force of low-sodium marinated braised beef (*p* < 0.05), and there was a significant interactive effect between the two factors (*p* < 0.05). As depicted in Figure 6, the shear force of low-sodium marinated braised beef in the L group was significantly lower than that in the C group throughout the storage period (*p* < 0.05). Both groups exhibited an initial increase in shear force followed by a decrease. The shear force of the L group reached its peak on day 3 and began to decline on day 6, whereas the shear force of the C group peaked on day 6 of storage.

#### 3.2.2. Exogenous L-Lys Addition on Meat Color of Low-Sodium Marinated Braised Beef

**Figure 7:**
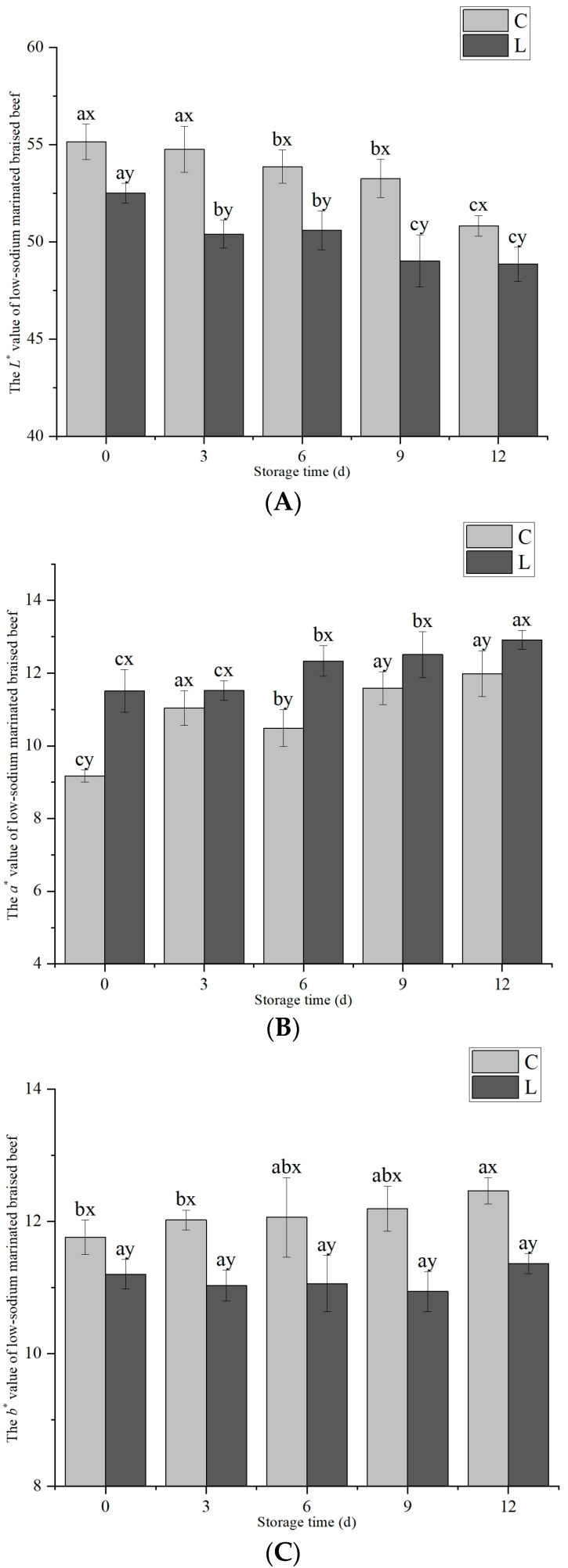
Different exogenous L-Lys treatments on L* (**A**), a* (**B**), and b* (**C**) values of low-sodium marinated braised beef during storage. L*, a*, and b* represent the lightness value, redness value, and yellowness value of the meat color. The letters ^a–c^ indicate significant differences (*p* < 0.05) among different storage times for the same exogenous L-Lys treatment. The letters ^x,y^ denote significant differences *(p* < 0.05) among different exogenous L-Lys treatments at the same storage time.

The supplementation of lysine and storage duration both exerted significant influences on the L*, a*, and b* values of low-sodium marinated braised beef (*p* < 0.05), and a notable interactive effect was detected between the two factors (*p* < 0.05). As illustrated in Figure 7, both exogenous L-Lys treatments and storage duration significantly influenced meat color (*p* < 0.05). The L* values of both low-sodium marinated braised beef groups decreased markedly with prolonged storage time (*p* < 0.05). Moreover, throughout the entire storage period, the L* value of the low-sodium marinated braised beef in the L group was significantly lower than that in the C group (*p* < 0.05), which was closely related to the moisture content. Specifically, the higher the moisture content, the lower the brightness. The a* values of both groups increased significantly over time (*p* < 0.05), except during the 3 d storage period. During the remainder of the storage period, the a* value of the L group was significantly higher than that of the C group (*p* < 0.05). The addition of L-Lys facilitated the chelation of ferrous ions, scavenging of free radicals, and delayed oxidation of myoglobin and lipids [22], thereby enhancing the a* value. Throughout the storage period, the b* value of the L group remained significantly lower than that of the C group (*p* < 0.05).

#### 3.2.3. Exogenous L-Lys Addition on Lipid Oxidation of Low-Sodium Marinated Braised Beef

**Figure 8:**
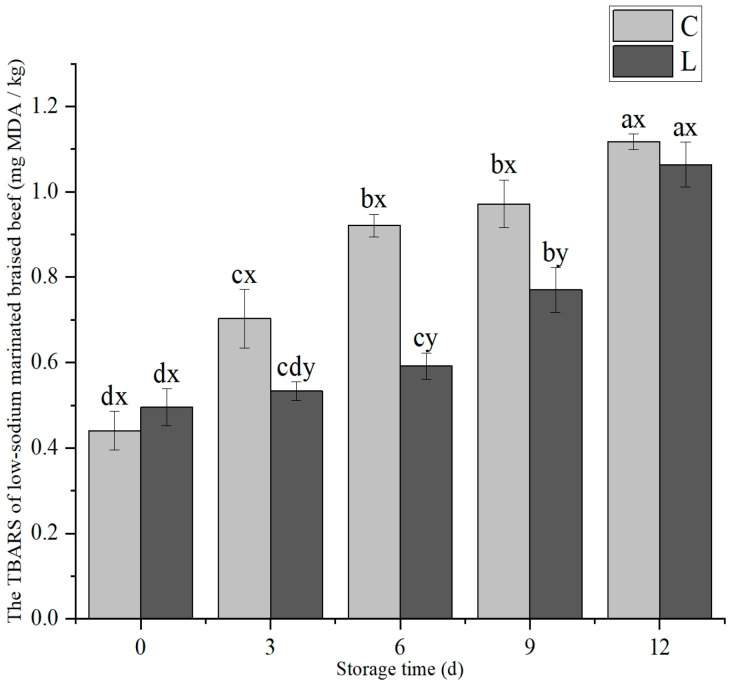
Exogenous L-Lys on TBARS value changes of low-sodium marinated braised beef during storage. The letters ^a–d^ indicate significant differences (*p* < 0.05) among different storage times for the same exogenous L-Lys treatment. The letters ^x,y^ denote significant differences (*p* < 0.05) among different exogenous L-Lys treatments at the same storage time.

The addition of lysine and storage time both had significant effects on the TBARS value of low-sodium marinated braised beef (*p* < 0.05), and there was a significant interaction between the two factors (*p* < 0.05). As presented in Figure 8, the TBARS values of both treatment groups demonstrated a significant increase with prolonged storage time (*p* < 0.05). Notably, during the storage period from 3 d to 9 d, the TBARS value of the L group was significantly lower than that of the C group (*p* < 0.05). When the storage time reached 12 d, the TBARS value of the L group rapidly increased to a level that was not significantly different from that of the C group *(p* > 0.05).

#### 3.2.4. Exogenous L-Lys Addition on the Total Viable Count (TVC) of Low-Sodium Marinated Braised Beef

**Figure 9:**
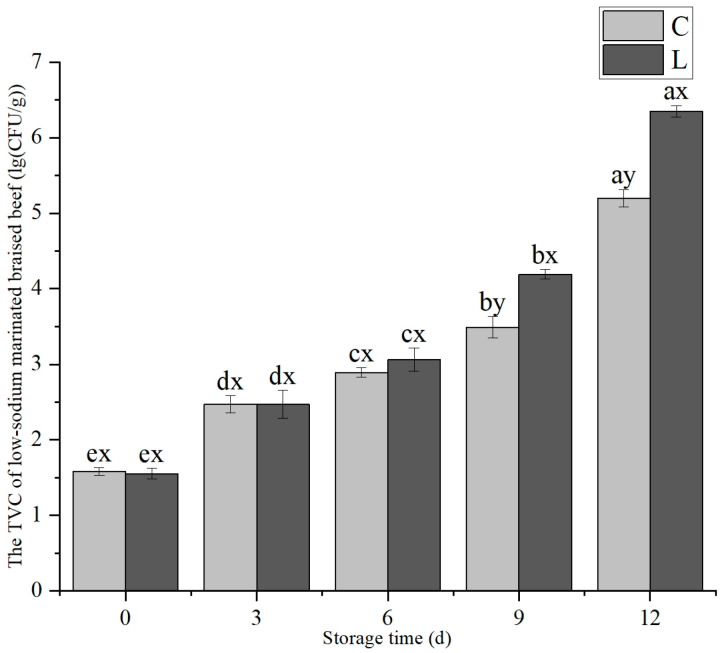
Exogenous L-Lys on the change in TVC in low-sodium marinated braised beef during storage. The letters ^a–e^ indicated significant differences (*p* < 0.05) among different storage times for the same exogenous L-Lys treatment. The letters ^x,y^ denote significant differences (*p* < 0.05) among different exogenous L-Lys treatments at the same storage time.

As shown in Figure 9, with the extension of storage time, the TVC of both the C group and L group marinated braised beef exhibited a significant exponential increase (*p* < 0.05). No significant difference in TVC was observed between the two groups during the initial storage period from 0 d to 6 d (*p* > 0.05). However, as storage progressed, at 9–12 d, the TVC of the L group significantly exceeded that of the C group (*p* < 0.05).

## 4. Discussion

The addition of exogenous L-lysine, an alkaline amino acid, caused an increase in pH, as the pH of 0.2–0.8% L-lysine was above 9. Moreover, the side-chain amino group (-NH_2_) of L-lysine neutralized acidic metabolites, such as lactic acid in muscle tissues, thereby increasing the pH value of the intracellular environment by Zhang et al. [3]. An increase in pH might enhance protein electrostatic repulsion and hydration; specifically, L-Lys could bind to myosin, enhancing its surface charge density and reducing the release of acidic substances caused by muscle tissue autolysis, thereby improving WHC and tenderness by Li et al. [23]. The addition of exogenous L-Lys effectively reduced the cooking loss of plain boiled beef by Wachirasiri et al. [24]. This might be attributed to the amino group (-NH_2_) of L-Lys facilitating the cross-linking of collagen and myosin through the formation of ε-lysine bonds, thereby constructing a more stable gel network and reducing the amount of moisture released during the cooking process by Nils et al. [25]. Furthermore, L-Lys could also enhance hydration by forming hydrogen bonds with water molecules through its hydrophilic groups, and it could alter the conformation of proteins to expose more hydrophilic sites [14,26]. Overall, the results presented here show that exogenous L-Lys addition can modify the structure of plain boiled beef via two separate mechanisms. One is through its direct, inherent actions, and the other is by changing the meat’s pH. These combined effects lead to an enhancement in the water holding capacity (WHC).

The addition of exogenous L-lysine also reduced the shear force of low-sodium plain boiled beef. Zhang et al. [3] found that the effects of L-arginine and L-Lys on the tenderness of chicken breast indicated that such alkaline regulatory effects not only influenced protein electrostatic repulsion, but also promoted hydration. Beef tenderness is regulated by factors such as salt-soluble protein solubility and myofibrillar protein phosphorylation. L-Lys has been shown to alter the secondary structure of myosin and enhance protein solubility; this process promotes the degradation of troponin-T and the breakdown of actomyosin, while inhibiting myosin aggregation, ultimately improving meat tenderness by Zhang et al. [3]. L-Lys achieved significant tenderization in low-sodium plain boiled beef by enhancing proteolytic enzyme activity, stabilizing cell membranes, and regulating osmotic pressure [27]. The addition of L-Lys significantly reduced the shear force of low-sodium plain boiled beef, improving its texture and palatability. The supplementation of exogenous L-lysine improved the meat color and appearance of low-sodium plain boiled beef. This might be attributed to the ability of L-Lys to chelate ferrous ions, thereby altering the protein structure of plain boiled beef by Ning et al. [28]. Concurrently, L-Lys promoted the solubility of salt-soluble proteins and enhanced WHC, thereby reducing the migration of moisture to the surface, which would otherwise lead to a decrease in the L* value of meat products. Moreover, L-Lys could inhibit lipid oxidation, thereby reducing the b* value [29,30]. Therefore, the addition of L-Lys could improve the color characteristics of low-sodium plain boiled beef, resulting in a more vivid red color and a more natural appearance.

Zhang et al. [3] found that L-Lys enhanced the electrostatic repulsion of proteins by increasing pH and promoted the dissociation of actomyosin, facilitating the transformation of free water into bound water and less mobile water. Additionally, L-Lys might also bind to myofibrillar proteins through its amino groups and promote collagen cross-linking, forming a stable three-dimensional network structure, thereby reducing water loss during cooking and storage by Wang et al. [31], significantly improved the WHC of low-sodium plain boiled beef (*p* < 0.05) and reduced cooking loss. Therefore, it was speculated that L-Lys might maintain the good quality of low-sodium plain boiled beef under low-salt conditions by optimizing the WHC and gel network structure of proteins and improving the distribution and state of water. Additionally, L-Lys could enhance the electrostatic repulsion of proteins by increasing the pH value, promote the precipitation of salt-soluble proteins, activate calcium-dependent proteases, promote the degradation of muscle proteins, release small molecular peptide chains, as described by Zhou et al. [23], form a stable gel network, and improve the microstructure of meat products, as described by Wang et al. [31]. Moreover, the amino group in L-Lys could form ionic bonds or hydrogen bonds with the carboxyl group of collagen, myosin, or other functional proteins, thereby softening the meat texture. However, if the concentration of L-Lys was too high, it might have led to excessive degradation of proteins, loss of elasticity, and even produced an undesirable texture, affecting the quality of meat products [21]. The addition of L-Lys causes an increase in the gap between muscle fibers, and this effect continuously enhanced with the increase in the addition amount. This result indicates that the addition of exogenous L-Lys might improve the tenderness and WHC of plain boiled beef by influencing its microstructure.

The addition of exogenous L-lysine effectively reduced the shear force of low-sodium marinated braised beef throughout the entire storage period because L-Lys has the function of delaying protein decomposition and water loss [23,32]. The addition of exogenous L-Lys reduced the shear force of low-sodium marinated braised beef and improved its tenderness. Moreover, L-Lys degrades troponin-T by maintaining the activity of calpain, thereby reducing the shear force and increasing the tenderness of the meat. Meanwhile, the addition of L-Lys could also increase the pH value of the meat, which also contributes to the improvement of the meat’s tenderness [3]. Exogenous L-Lys delayed the color deterioration of low-sodium marinated braised beef during storage by improving the WHC and antioxidant capacity. The addition of L-Lys could scavenge DPPH radicals and hydroxyl radicals [33], enhance the antioxidant property of low-sodium marinated braised beef, and retard the oxidation of myoglobin and lipids [23]. As a result, the color of the low-sodium marinated braised beef in the L group was more stable during the shelf life, enabling the product to exhibit a more vivid and natural red characteristic.

With the increase in storage time, the degree of fat oxidation in low-sodium marinated braised beef continuously increased. The addition of exogenous L-Lys was helpful to alleviate the rate of fat oxidation in meat products in the early stage of storage. However, when the storage time reached day 12, this effect became ineffective. Moreover, as the storage time extended, the total number of colonies in the beef gradually increased. This suggested that it might be because the increase in bacteria decomposed the fat, which not only caused the pH value to rise, but also promoted the fat oxidation of the beef, as reported by Krochmal-Marczak et al. [34]. The study and analysis of the total number of colonies in low-sodium marinated braised beef showed that the effects of exogenous L-Lys on meat products are not entirely beneficial. The lower salt content in meat is more conducive to bacterial growth. During the shelf life, the pH values of meat and meat products might change because of microbial activity [35]. This change has the potential to affect the product storage performance. The total viable count (TVC) exhibited exponential growth in the late storage period, and both groups showed spoilage at 12 days of storage; thus, the addition of exogenous L-Lys had no effect on the shelf life of low-sodium marinated braised beef. This experiment investigated the effects of exogenous L-lysine on improving the quality characteristics of low-sodium plain boiled beef and low-sodium marinated braised beef. However, it failed to conduct detailed studies on the action mechanism of L-lysine on beef, protein structural changes, and color variations during shelf life, which need to be verified by follow-up research.

## 5. Conclusions

This experiment confirmed the hypothesis proposed in the introduction that L-lysine exerted improving effects on the quality, appearance, and microstructure of low-sodium plain boiled beef. Moreover, it enhanced the edible quality of low-sodium marinated braised beef without compromising its shelf life. When the KCL substitution rate is 60%, the quality of low-sodium plain boiled beef and low-sodium marinated braised beef can be improved by adding 0.6% exogenous L-Lys. In low-sodium plain boiled beef, L-Lys effectively mitigated the deterioration of meat quality induced by low-sodium processing through increasing pH, reducing cooking loss, elevating a* value, and decreasing shear force (*p* < 0.05). Additionally, the optimal addition level of 0.6% was determined by combining sensory evaluation. The marinated braised beef was prepared from plain boiled beef treated with 0.6% L-Lys. During the 0–12 d storage period, the a* value of the marinated braised beef was progressively increased, resulting in a more vivid red color. Moreover, its tenderness and fat oxidation stability were improved. Although the TVC from 9 to 12 d was higher in the low-sodium group with L-Lys addition compared to the low-sodium group without L-Lys, both groups had spoiled by 12 d; thus, the L-Lys addition did not affect the product shelf life. The addition of L-Lys primarily optimizes beef quality by enhancing hydration, improving muscle structural properties, and exerting antioxidant synergistic effects. Thus, the addition of exogenous L-Lys provided critical theoretical support and technical guidance to produce low-sodium plain boiled beef and its further-processed derivatives. Future research could investigate the synergistic interactions between L-Lys and other amino acids to achieve a holistic improvement in product quality.

## Figures and Tables

**Table 1:** Different addition levels of L-Lys on the quality of low-sodium plain boiled beef.

Index	Exogenous L-Lys Addition (%)				
	0	0.2	0.4	0.6	0.8
pH	5.89 ± 0.05 ^e^	6.08 ± 0.05 ^d^	6.25 ± 0.08 ^c^	6.36 ± 0.08 ^b^	6.45 ± 0.07 ^a^
Cooking loss	40.50 ± 0.79 ^a^	37.79 ± 1.18 ^b^	35.51 ± 1.29 ^c^	34.08 ± 0.67 ^d^	31.86 ± 0.96 ^e^
Shear force	71.35 ± 6.06 ^a^	53.00 ± 7.08 ^b^	39.75 ± 7.63 ^c^	28.57 ± 4.90 ^d^	27.59 ± 3.91 ^d^
L*	55.70 ± 0.78 ^a^	54.40 ± 0.13 ^b^	52.46 ± 0.72 ^c^	50.11 ± 0.85 ^d^	47.95 ± 0.98 ^e^
a*	9.01 ± 0.92 ^e^	9.78 ± 0.82 ^d^	10.35 ± 0.65 ^c^	11.32 ± 0.74 ^b^	12.35 ± 0.66 ^a^
b*	12.23 ± 0.38 ^a^	11.98 ± 0.26 ^ab^	11.60 ± 0.31 ^b^	10.85 ± 0.39 ^c^	9.98 ± 0.79 ^d^
C*	15.21 ± 0.75 ^b^	15.28 ± 0.51 ^b^	15.61 ± 0.33 ^ab^	15.66 ± 0.60 ^ab^	16.29 ± 0.86 ^a^
H*	0.94 ± 0.04 ^a^	0.91 ± 0.07 ^ab^	0.84 ± 0.04 ^b^	0.77 ± 0.04 ^c^	0.71 ± 0.07 ^c^
*W*	52.96 ± 0.95 ^a^	52.68 ± 1.55 ^a^	50.49 ± 1.17 ^b^	47.25 ± 0.69 ^c^	46.35 ± 1.85 ^c^

L*, a*, b*, C*, H*, and *W* represent the lightness value, redness value yellowness chroma, and hue value of meat color. Means with different superscripts (^a–e^) were significantly different (*p* < 0.05).

**Table 2:** Different L-Lys addition levels on relaxation time of low-sodium plain boiled beef.

Index	Exogenous L-Lys Addition (%)				
	0	0.2	0.4	0.6	0.8
*T*_21_	3.08 ± 0.50 ^a^	3.61 ± 0.05 ^ab^	2.37 ± 0.20 ^ab^	2.81 ± 0.57 ^ab^	2.67 ± 0.58 ^ab^
*T*_22_	90.55 ± 2.29 ^e^	90.96 ± 0.18 ^d^	92.71 ± 0.79 ^c^	93.12 ± 1.33 ^b^	93.48 ± 1.33 ^a^
*T*_23_	6.37 ± 0.39 ^d^	5.44 ± 0.15 ^d^	4.60 ± 0.11 ^c^	4.07 ± 0.48 ^b^	3.85 ± 0.31 ^a^

Means with different superscripts (^a–e^) were significantly different (*p* < 0.05).

## Data Availability

The original contributions presented in the study are included in the article, and further inquiries can be directed to the corresponding author.
